# Lymphoepithelial carcinoma in the maxillary sinus: a case report

**DOI:** 10.1186/1752-1947-6-416

**Published:** 2012-12-11

**Authors:** Darouichi Mohammed, Alshammari Jaber, Monnier Philippe, Sandu Kishore

**Affiliations:** 1 Department of Radiology, Hôpital Neuchatelois, La Chaux-de-Fonds, Switzerland; 2 Department of ORL and Head and Neck surgery, CHUV, Lausanne, Switzerland; 3 Department of ORL and Head and Neck surgery, L’Hôpital du Valais, Center Hospitalier du Center du Valais-CHCV, 1950, Sion, Switzerland

**Keywords:** Maxillary sinus, Lymphoepithelial carcinoma

## Abstract

**Introduction:**

Lymphoepithelial carcinoma of the maxillary sinus is a very rare malignancy and it can be difficult to make a pre-operative diagnosis.

**Case presentation:**

A 72-year-old Caucasian woman presented to our facility with an isolated right-side epistaxis that had been present for three months, with the results of a computed tomography scan showing a soft tissue mass in the right maxillary sinus with an impacted tooth. The results of a transnasal endoscopic biopsy were compatible with a lymphoepithelial carcinoma, following which our patient underwent a radical excision of the mass. The final histology results revealed lymphoepithelial carcinoma of the maxillary sinus with negative assays for Epstein-Barr virus. Our patient was given post-operative external radiotherapy and has remained disease-free at three-year follow-up.

**Conclusions:**

This report details the diagnosis and management of a case of lymphoepithelial carcinoma of the maxillary sinus, which is a very rare malignant tumor with very little mention in the literature. Only a strong suspicion with systematic use of various patho-immunological tests helps to arrive at a definitive diagnosis by excluding other better-known tumors.

## Introduction

A majority of malignant tumors of the paranasal sinuses occur in the maxillary sinus [1,2]. Among malignant lesions of the maxillary sinus, lymphoepithelial carcinoma is very rare. Lymphoepithelial carcinoma is characterized by nests, sheets, or individual undifferentiated or poorly differentiated malignant epithelial cells surrounded and infiltrated by prominent components of small, mature lymphocytes and plasma cells [3]. The more frequent sites of these tumors are the nasopharynx, salivary glands and the larynx [4]. Very few cases of lymphoepithelial carcinoma arising in the maxillary sinus have been reported [5,6] with none precisely detailing its diagnostic characteristics and management. In this report we present a case of maxillary sinus lymphoepithelial carcinoma and review the radiological findings, macroscopic and microscopic features, immunohistology and the treatment options available.

## Case presentation

**Figure 1:**
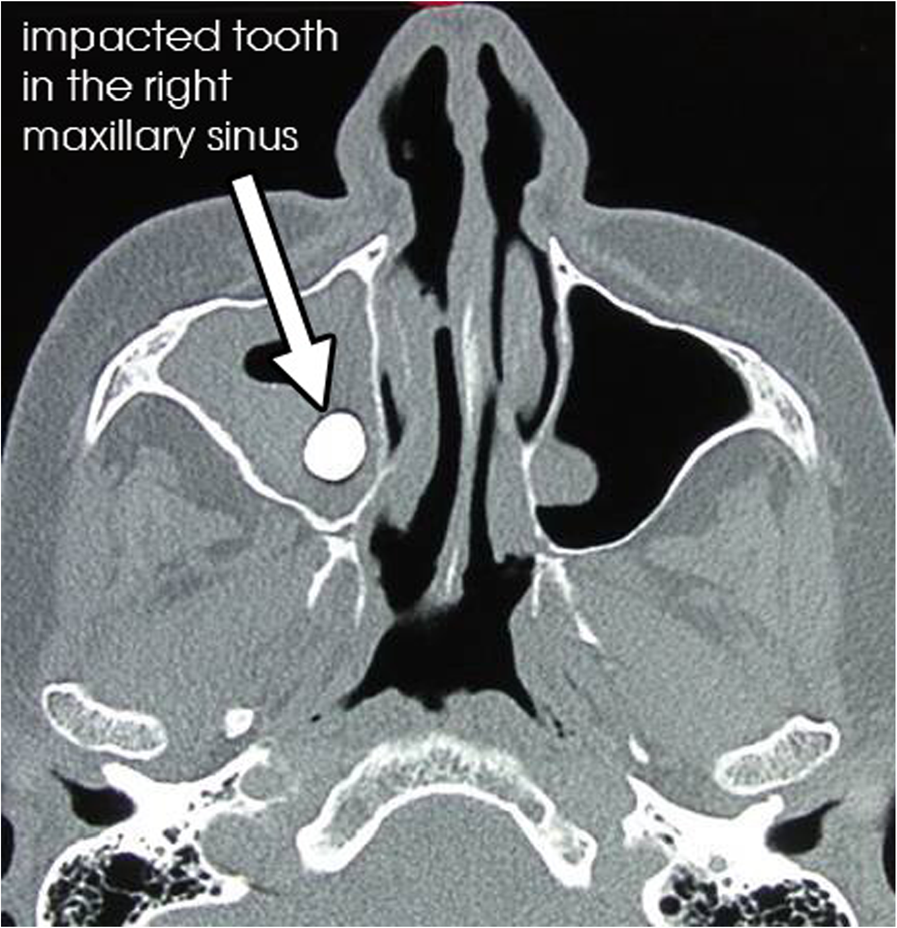
Axial computed tomography image showing a homogenous soft tissue mass occupying the entire right maxillary sinus with an impacted tooth.

A 72-year-old Caucasian woman presented to our facility with an isolated right-side epistaxis that had been present for three months without other associated otolaryngological, ophthalmological or neurological symptoms. There was no history of trauma or chronic sinonasal infections. Ten years prior to the incident, our patient was diagnosed as having breast cancer and was treated with surgery and chemoradiotherapy. The results of a physical examination were normal except for a thread of blood coming out of the right middle meatus. A neck examination revealed no palpable lymph nodes. Routine laboratory investigation results were normal. A paranasal sinus (PNS) computed tomography (CT) scan showed expansion of the right maxillary sinus by a homogenous soft tissue mass without calcification, occupying the entire sinus cavity. The sinus walls were intact with no bony erosion. There was an impacted tooth visible inside the mass (Figure 1). Other sinus cavities were normal. Endoscopic maxillary sinus meatotomy with tissue biopsy revealed carcinoma of the lymphoepithelial type. Our patient underwent a lateral rhinotomy with an orbital floor and hard palate saving maxillectomy, with complete excision of the tumor.

**Figure 2:**
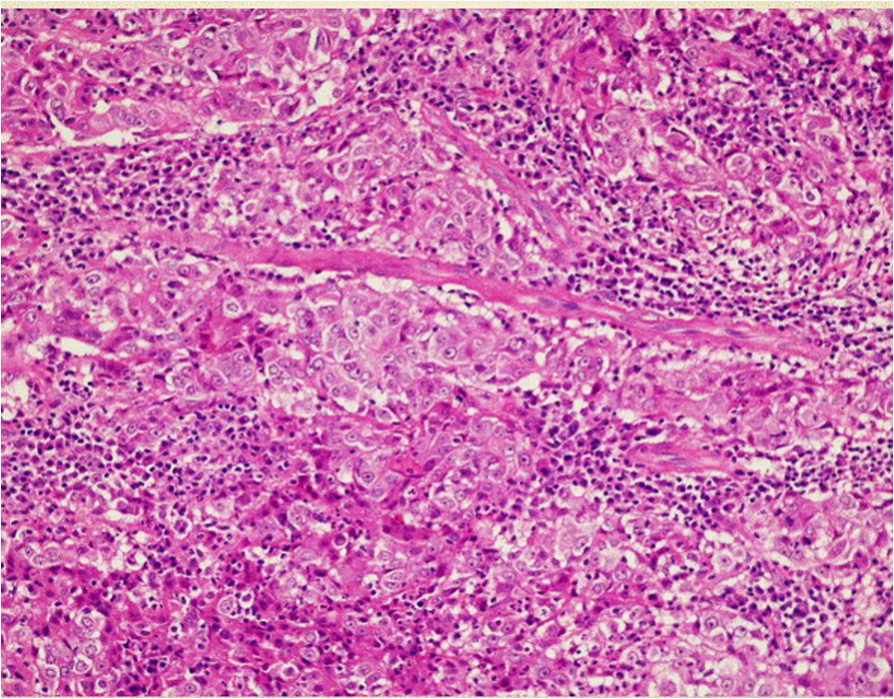
Histological slide showing malignant epithelial cells with large oval vesicular nuclei containing fine chromatin, prominent eosinophilic nucleoli and large quantities of lymphocytes and plasma cells.

**Figure 3:**
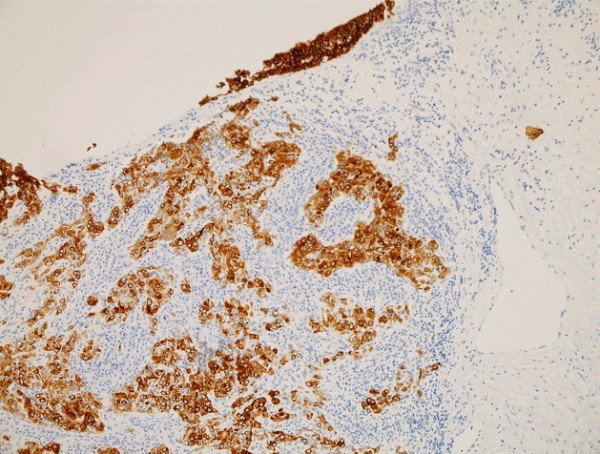
Histological slide showing malignant epithelial cells with a strong positivity for proliferative marker Ki-67 (MIB-1).

Histopathological examination (Figure 2) revealed a poorly differentiated carcinoma composed of malignant epithelial cells having large oval vesicular nuclei and containing fine chromatin with prominent one to three eosinophilic nucleoli. The tumor showed large quantities of lymphocytes and plasma cells. Immunohistochemical staining revealed that these cells were positive for pancytokeratin marker (MNF 116), proving the epithelial nature of these cells. Tests for cytokeratin (CK) 5/6 were mildly positive and favored squamous differentiation, while CK7 and carcinoembryonic antigen (CEA) tests were negative. By performing these investigations, we excluded a poorly differentiated squamous carcinoma. Melanin marker studies excluded a malignant melanoma. A CK20 antigen test result was negative, thus excluding an intestinal type of adenocarcinoma. There was a strong positivity for the proliferative marker Ki-67 (MIB-1) (Figure 3). Results of hybridization assays for Epstein-Barr virus were negative. Thus, the immunohistochemical studies were diagnostic for a lymphoepithelial carcinoma. External radiotherapy with a cumulative dose of 48Gy was delivered post-operatively. Our patient showed satisfactory treatment response and has been followed up very closely over the last three years. She has remained free of disease with no locoregional or distant metastasis.

## Discussion

Lymphoepithelial carcinoma of the maxillary sinus is a very rare malignant tumor and was described for the first time by Schminke and Regaud in 1921 [7]. The lymphoepithelial carcinoma frequently develops in the nasopharynx, salivary glands and the larynx. More rarely, it affects the lungs, esophagus, stomach, pancreas, skin, cervix, endometrium, vulva, kidney, bladder, and central nervous system [8]. In the majority of cases, patients are asymptomatic, and the lesion is discovered incidentally by imaging [9]. Otherwise, patients complain of non-specific sinonasal symptoms or may have obstructive nasal symptoms related to acute or chronic maxillary sinusitis [6] and rarely present with epistaxis, as in our patient. There is a strong association with an Epstein-Barr virus (EBV) infection [10]. Interestingly, patients with lymphoepithelial carcinoma in Western Europe and the USA are usually Epstein-Barr virus negative [11]. According to Lezzoni *et al*. the presence or absence of Epstein-Barr virus in the lymphoepithelial carcinoma of the maxillary sinus does not have any prognostic importance [12].

The lymphoepithelial carcinoma developing from the maxillary sinus can present as an aggressive tumor with signs of local invasion to nerves, orbit, or the rhinopharynx [13]. In general, the routine laboratory tests are normal. In terms of imaging, the lymphoepithelial carcinoma of the maxillary sinus appears as a diffuse opacity of soft tissue density on standard radiography [14]. Paranasal sinus CT scan highlights a solid homogenous mass occupying the maxillary sinus cavity that is not enhanced with an intravenous contrast injection [15]. In our patient’s case, she had an impacted tooth inside the mass. There are no pathognomic radiological criteria that differentiate the lymphoepithelial carcinoma from other tumors of the maxillary sinus, especially squamous cell carcinomas or lymphomas. All these three tumors are locally destructive and have metastases to retropharyngeal and cervical nodes [11,15]. In general, clinicoradiological tests are non-contributory to the final diagnosis. Histology and immunohistochemical analysis establish the definitive diagnosis of a lymphoepithelial carcinoma. The microscopic features of this tumor show epithelial cells with eosinophilic cytoplasm. Their large oval nuclei have fine vesicular chromatin with one to three prominent red nucleoli. The fibrous stroma is heavily infiltrated by plasma cells and lymphocytes. Immunohistochemical staining shows that epithelial cells of lymphoepithelial carcinoma stain positively for pancytokeratin marker (MNF 116) and MIB-1 and stain negatively for CK 5/6, CK 7, and CEA. There is no immune reactivity for melanin A marker and CK 20.

Because of the limited number of cases, there is no standardized treatment policy reported to date for lymphoepithelial carcinoma of the maxillary sinus [3]. The initial treatment for maxillary sinus tumors has always been surgery. Lymphoepithelial carcinoma is known to be radiosensitive and hence we decided on a course of post-operative radiotherapy for our patient [14]. Adjuvant chemotherapy and neck management has been suggested for advanced and extensive disease [3]. Our patient did not receive any chemotherapy and neck management as she did not have neck metastasis. The rate of recurrence in maxillary sinus lymphoepithelial carcinoma is reported to be roughly 25 percent [15]. A regular follow-up with periodic CT scans is recommended.

## Conclusions

Lymphoepithelial carcinoma of the maxillary sinus is a very rare malignant tumor with little mention in the literature. Its pre-operative diagnosis is difficult and the final definitive diagnosis is established by histopathological examination and immunohistochemical studies. The treatment consists of surgery and radiotherapy with the possibility of achieving acceptable cure rates. A strong suspicion and systematic use of various immunological tests helps to arrive at a definitive diagnosis by excluding other better-known tumors.

## Consent

Written informed consent was obtained from the patient for publication of this case report and any accompanying images. A copy of the written consent is available for review with the Editor-in-Chief of this journal.

## Competing interests

The authors declare that they have no competing interests in the preparation of this article.

## Authors’ contributions

DM, collection of data, manuscript preparation; AJ, collection of data, manuscript preparation; MP, manuscript preparation, analysis; SK, manuscript preparation, analysis. SK was the major contributor to writing the manuscript. All authors read and approved the final manuscript.
